# RNA-DNA differences in variant calls from cattle tissues result in erroneous eQTLs

**DOI:** 10.1186/s12864-024-10645-z

**Published:** 2024-08-01

**Authors:** Alexander S. Leonard, Xena M. Mapel, Hubert Pausch

**Affiliations:** Animal Genomics, ETH Zurich, Universitaetstrasse 2, Zurich, 8092 Switzerland

**Keywords:** RNA variant calling, RNA DNA differences, eQTL, Livestock genetics, Pseudogenes

## Abstract

**Background:**

Association testing between molecular phenotypes and genomic variants can help to understand how genotype affects phenotype. RNA sequencing provides access to molecular phenotypes such as gene expression and alternative splicing while DNA sequencing or microarray genotyping are the prevailing options to obtain genomic variants.

**Results:**

We genotype variants for 74 male Braunvieh cattle from both DNA (~ 13-fold coverage) and deep total RNA sequencing from testis, vas deferens, and epididymis tissue (~ 250 million reads per tissue). We show that RNA sequencing can be used to identify approximately 40% of variants (7–10 million) called from DNA sequencing, with over 80% precision. Within highly expressed coding regions, over 92% of expected variants were called with nearly 98% precision. Allele-specific expression and putative post-transcriptional modifications negatively impact variant genotyping accuracy from RNA sequencing and contribute to RNA-DNA differences. Variants called from RNA sequencing detect roughly 75% of eGenes identified using variants called from DNA sequencing, demonstrating a nearly 2-fold enrichment of eQTL variants. We observe a moderate-to-strong correlation in nominal association p-values (Spearman ρ^2^ ~ 0.6), although only 9% of eGenes have the same top associated variant.

**Conclusions:**

We find hundreds of thousands of RNA-DNA differences in variants called from RNA and DNA sequencing on the same individuals. We identify several highly significant eQTL when using RNA sequencing variant genotypes which are not found with DNA sequencing variant genotypes, suggesting that using RNA sequencing variant genotypes for association testing results in an increased number of false positives. Our findings demonstrate that caution must be exercised beyond filtering for variant quality or imputation accuracy when analysing or imputing variants called from RNA sequencing.

**Supplementary Information:**

The online version contains supplementary material available at 10.1186/s12864-024-10645-z.

## Background

High-throughput RNA sequencing (RNA-seq) has been frequently applied for measuring gene expression levels [1], assembling *de novo* transcriptomes [2], detecting copy number alterations [3], and identifying genomic variants that influence gene expression [4]. Genotypes called from RNA-seq have also been used to determine population structure [5, 6]. Historically, RNA-seq has been viewed as unreliable input compared to DNA-seq for identifying genetic variation. Whole-genome DNA-seq based studies often use between 200 and 500 million reads (corresponding to approximately 10-fold to 25-fold coverage of a mammalian genome), while RNA-seq based studies aim for between 30 million reads to measure gene expression and 100 million reads to quantify alternative splicing events and map splicing QTL (sQTL) [7]. Messenger RNA (mRNA) is the most common source for RNA-seq, primarily containing coding and untranslated regions within genes which only represent a small fraction of the genome. Conversely, total RNA-seq contains greater amounts of noncoding RNA and non-polyadenylated transcripts [8], representing more of the transcriptome, but incurs higher cost than mRNA-seq.

RNA-seq variant callers are less common than their DNA-seq counterparts, but GATK [9] and a combination of preprocessing RNA-seq reads with Opposum [10] and calling variants with Platypus [11] have been the dominant options. Recently, DeepVariant has been extended to provide an RNA-seq trained model [12], greatly improving the accuracy and quantity of variants called from RNA-seq compared to the previous state of the art. The improved DeepVariant model also reduces the number of variants called at sites subjected to A-to-I editing within the RNA-seq. Such RNA editing events warrant attention as they can have important functional effects [13].

As a consequence of these factors, as well as expression variability in different tissues, far fewer genetic variants are called with RNA-seq than DNA-seq, with earlier studies identifying only 100k variants from 7 cow transcriptomes [14] or 68k variants from 29 cow transcriptomes [5]. Even large studies like the cattle Genotype-Tissue Expression (GTEx) project [15], could only confidently call 22k variants from 7,180 publicly available transcriptomes of diverse origin, which is several orders of magnitude less than called from similar sized cohorts with WGS data [16]. These variants can then be imputed to higher density using large reference panels, like that of the 1000 Bull Genomes project [16]. However, a strong depletion of non-coding variants in typical RNA-seq datasets results in a less reliable imputation of variants that are distant to transcribed regions. Similar observations have been made in chicken [17], pig [18], and human [19].

The mapping of expression and splicing quantitative trait loci (e/sQTL) is increasingly performed to investigate the impact of regulatory regions on phenotypes. These loci can be detected through association testing between molecular phenotypes (e.g., gene expression and splicing levels quantified from RNA-seq) and genetic variation. Recent studies have identified e/sQTL in cattle affecting economically relevant traits, such as male fertility [4], milk production [20], and carcass yield [21]. These e/sQTL have proven highly valuable in prioritizing candidate causative variants for complex traits and diseases [15, 22].

In this work, we reanalyse deeper-than-usual (~ 250 million reads) total RNA sequencing across three tissues in a subset of 74 cattle samples previously analysed for e/sQTL using DNA-seq derived genotypes [4]. We compare variants called with DeepVariant from DNA-seq and RNA-seq from each tissue and examine RNA-DNA differences. These RNA-seq based variant calls are enriched for eQTL and their nominal p-values are strongly correlated with those from the WGS-derived eQTL. Even as RNA-seq coverage is subsampled down to 100 and 30 million reads, we still observe strong variant calling precision and recall.

## Methods

### DNA and RNA alignment

We considered 74 bulls with publicly available whole-genome DNA and total RNA (including ribosomal depletion steps) sequence data from three male reproductive tissues previously used to characterize gene expression and splicing variability [4] (Supplementary Table 1). Adapter sequences and low-quality bases were trimmed from all DNA and RNA reads, while poly-A/G tails were filtered from RNA reads with fastp (v0.23.4) [23]. The DNA-seq data were aligned to the cattle reference (ARS-UCD1.2) with bwa-mem2 (v2.2.1) [24, 25] with the flag “-M”. The alignments were deduplicated and sorted with SAMtools (v1.19.1) [26]. RNA-seq reads were aligned to the same reference and the Ensembl gene annotation (v108) using the splice-aware aligner STAR (v2.7.9) [27] with --waspOutputMode and heterozygous SNPs from DNA to account for allelic imbalance. Read depth was estimated with perbase (v.0.8.5) (https://github.com/sstadick/perbase) and coverage per annotation classification was calculated with bedtools (v2.30) annotate [28] using the Ensembl v108 annotation.

Lower sequencing coverage was simulated by downsampling with SAMtools view -s < fraction>, where the fraction was chosen to approximately sample one hundred, thirty, and five million paired-end read subsamples.

### Variant calling and analysis

Variants were called from the aligned bam files using DeepVariant (v1.5) [29]. For the DNA samples, we additionally used the “insert_size” channel, while for the RNA samples we used “--split_skip_reads” and the v1.4 RNA checkpoint model. All samples for each set of DNA or RNA tissue were merged using GLnexus (v1.4.1) [30]. Sporadically missing genotypes were imputed using Beagle (v4.1) [31] using the “gl” field. For analyses explicitly referencing an external reference panel, we imputed variants with Beagle (v5.4) using the “gt” field and an existing reference panel containing 501 cattle [32]. Variant call intersection sets were calculated with BCFtools (v1.19) [26] isec. Precision/recall/F1 were calculated with hap.py (v0.3.15) (https://github.com/Illumina/hap.py), stratifying by region with a bed file containing annotated exon coordinates based on their expression level quantified in transcripts per million (TPM).

Principal components (PCs) were calculated with plink2 (v2.00a4LM) [33], using a minimum allele frequency of 5% and treating half calls as missing. Each individual’s breed was assigned according to the Swiss Braunvieh herdbook. Variant effects were classified with VEP (v108) [34], using the flags ‘--tab --fields “Consequence, IMPACT” --species bos_taurus’. Regions without variants were identified by converting VCF to BED format, followed by merging blocks within 1 Kb of each other using BEDtools merge -d 1000. We then assessed uncovered regions using BEDtools genomecov.

Allele-specific expression was calculated on the WASP filtered alignments with QTLtools (v1.3.1) [35] with the ase command and the “--both-alleles-seen” flag to remove monoallelic expression.

### eQTL analysis

We used QTLtools quan to estimate gene expression in transcripts per million (TPM) and featureCounts (v2.0.4) [36]. We included genes with ≥ 0.1 TPM in ≥ 20% of samples and ≥ 6 reads in ≥ 20% of samples, and quantile normalised the expression values. Principal components for LD-pruned variant calls and RNA expression were calculated with QTLtools pca.

We split multiallelic variants into multiple biallelic variants, then removed sites with < 1% minor allele frequency using BCFtools. We identified eQTL within 1 Mb of the transcription start site with QTLtools and the “--normal” flag. Bull age, RNA integrity number, the first 3 genotype PCs, and the top 10 PCs of the TPM matrix were used as fixed covariates. We performed 1000 permutations and used a false discovery rate of 5% to estimate per-gene significance thresholds, followed by a conditional pass to estimate independent eQTL signals.

Specific eQTL and nearby variants were visualised from alignment and variant call files with IGV (v2.17.4) [37].

## Results

### RNA sequencing alignment

**Fig. 1:**
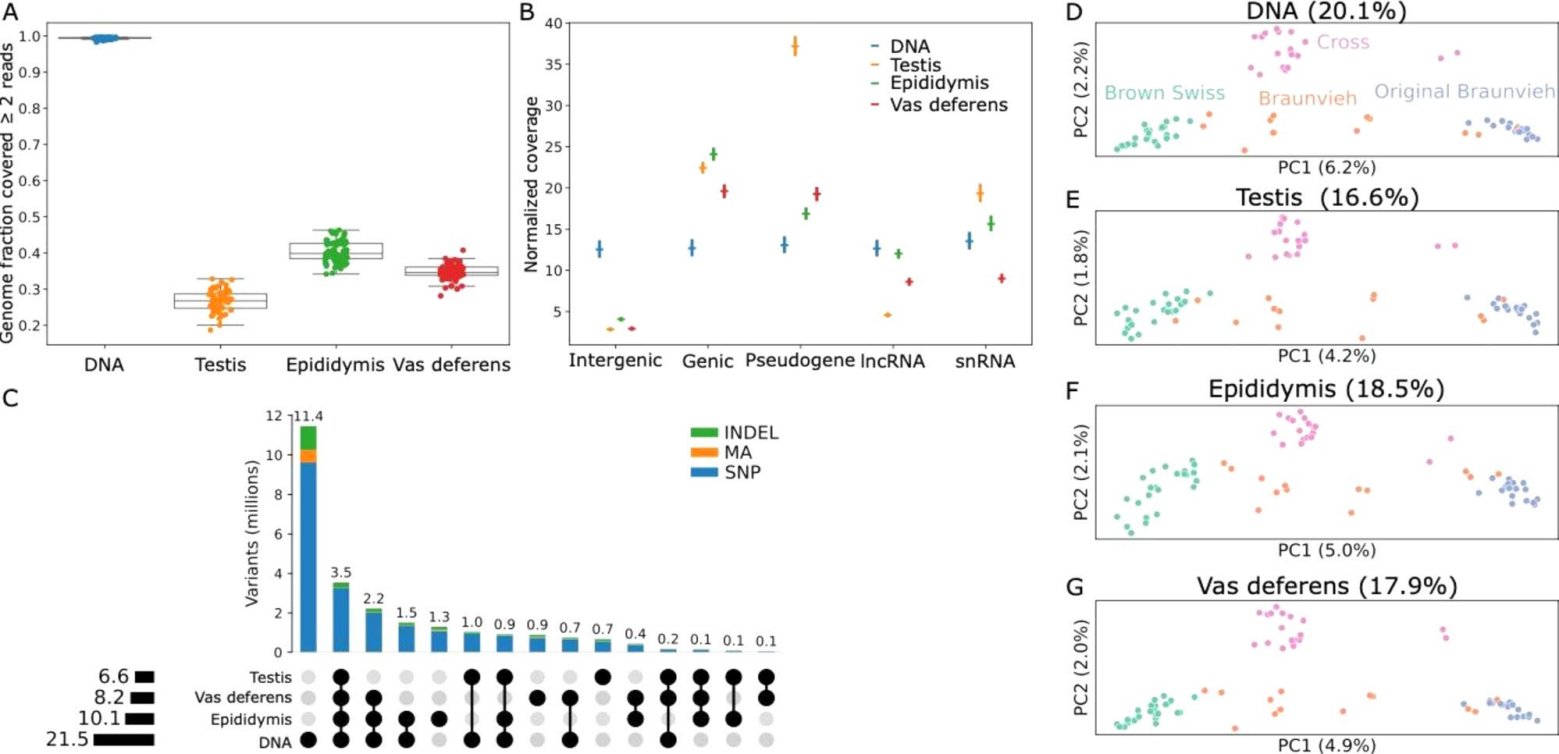
Alignment and variant calling from DNA and RNA sequencing data. (**A**) Fraction of the autosomal bases covered by at least two reads for DNA-seq and the three RNA-seq tissues. (**B**) Coverage depth normalised by the total size of the features across different annotated regions for DNA and the three RNA tissues. Intergenic regions have low coverage in RNA-seq while other categories are enriched, like long noncoding (lnc) and small nuclear (sn) RNA. DNA coverage is consistent across all categories. (**C**) Overlap of called variants based on exact position and REF/ALT matches, stratified by SNPs, indels, and multiallelic (MA) variants. (**D**-**G**) Principal component analyses using variants called from DNA-seq or RNA-seq from one of the three tissues. Braunvieh refers to animals of ambiguous or mixed Brown Swiss or Original Braunvieh ancestry, and cross refers to Brown Swiss or Original Braunvieh crossed with a different breed. The percentages above each plot refer to the total variance explained by the top 10 principal components

We considered 74 mature Braunvieh bulls that had DNA-seq as well as total RNA-seq from testis, epididymis, and vas deferens tissues [4]. The mean sequencing coverage for DNA was 13.3 ± 3.9-fold (approximately ~ 240 million reads). The mean RNA-seq coverage for testis, epididymis, and vas deferens tissues was 258 ± 33, 284 ± 36, 263 ± 24 million reads, respectively. After aligning reads to the ARS-UCD1.2 bovine reference genome, an average of 99.6% of the autosomal bases were covered by at least 2 reads with DNA-seq, while for testis, epididymis, and vas deferens RNA-seq the average was 26.7%, 40.4%, and 34.8%, respectively (Fig. 1A). Coverage of the DNA-seq reads was even across different annotated regions of the reference genome while the coverage of RNA-seq reads was strongly enriched in genic regions (Fig. 1B; Supplementary Fig. 1). As expected for total RNA-seq, we also identified elevated coverage in regions overlapping miscellaneous micro/small/long noncoding RNA that are not typically enriched in mRNA-seq. We observed moderate coverage in intergenic regions, which is likely due to the incomplete annotation of the bovine genome [38], particularly of long noncoding RNAs or underrepresented tissue-specific genes.

We used DeepVariant to call variants for each sample on the DNA and each RNA tissue type separately. Across the autosomes, there were 21.5 M called variants for DNA and 6.6 M, 8.2 M, and 10.1 M variants for testis, vas deferens, and epididymis RNA, respectively. Compared to the number of variants called from DNA-seq, the number of variants called from each tissue was nearly proportional to the fraction of the genome covered by RNA-seq for that tissue, respectively 31%, 38%, and 47%. This suggests that RNA-seq can be used to call variants at a similar rate to DNA sequencing wherever there is sufficient coverage. The DNA-seq variants were more evenly distributed across the genome compared to RNA-seq variants, with almost 96% of autosomal bases within 1 Kb of a DNA-seq variant compared to 64–75% for RNA-seq variants (Supplementary Fig. 2), implying regions of the genome remain completely inaccessible from total RNA sequencing.

The ratios of transitions to transversions (Ti: Tv) for the total RNA-seq variants ranged from 2.19 in non-exonic or noncoding exons to 3.58 within coding exons (Table 1), broadly in line with the distinct expectations for genome-wide or the more conserved coding regions [39]. Most DNA-specific variants were in intergenic regions, where there was less RNA coverage. RNA variants within intergenic regions largely behaved as expected, although the increased Ti: Tv for epididymis and vas deferens may result from tissue-specific genes that are not yet correctly annotated. Using DNA-seq also resulted in proportionally increased indel calls, accounting for 14% of variant calls compared to ~ 11% in total RNA-seq, as well as multiallelic calls (3.4% in DNA-seq versus ~ 1.3% in total RNA-seq). Approximately 3.5 million variants were present in all four datasets, indicating a large portion of regions are all expressed across the three examined tissues. RNA genotypes from the three tissues captured the same population structure as the DNA (Fig. 1D-G), demonstrating that the RNA variant calls contained meaningful variation.

**Table 1:** Median number of biSNPs (biallelic SNPs) in coding exons, noncoding exons (e.g., pseudogenes, lncRNA, etc.) and non-exon regions per sample with the associated Ti: tv rate for variants called from DNA-seq or the three RNA-seq tissues

	Coding exons		Noncoding exons		Not exons	
	biSNPs	Ti: Tv	biSNPs	Ti: Tv	biSNPs	Ti: Tv
DNA	39,657	3.13	10,113	2.16	6,652,085	2.20
Testis	35,458	3.53	5,669	2.19	1,427,805	2.20
Epididymis	34,396	3.53	6,082	2.21	2,265,030	2.41
Vas deferens	31,913	3.58	5,409	2.25	1,933,053	2.43

We used the variant effect predictor (VEP) to assess potential consequences for the called variants. The RNA-seq proportionally called more variants annotated as low/moderate/high impact (Supplementary Fig. 3), with the strongest enrichment (nearly 2-fold) observed in testis. On average across the tissues, between 70 and 75% of low/moderate/high impact variants called from DNA were present in the RNA variants, again suggesting the RNA called variants are primarily missing intergenic variants for which functional consequences are not immediately apparent.

### RNA variant calling accuracy

**Fig. 2:**
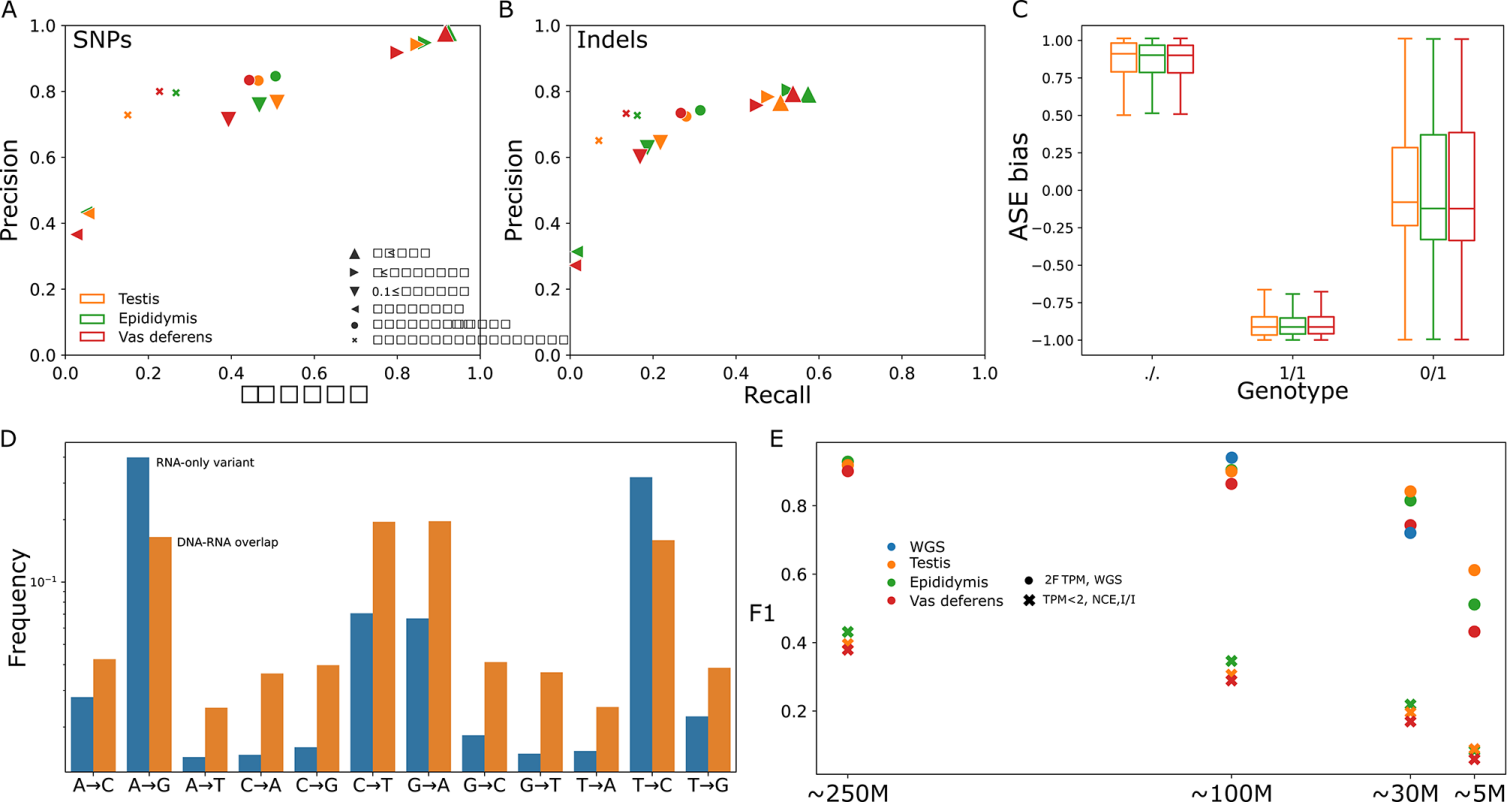
Variant precision and recall for SNPs (**A**) and indels (**B**) called from RNA-seq using DNA-seq variants as truth, stratified by non-exonic, noncoding exons, and different levels of coding exon expression. (**C**) Heterozygous variants misgenotyped as homozygous reference/missing or homozygous alternate displayed strong allelic imbalance, where positive (negative) ASE indicates the reference allele was more (less) expressed. Outliers are not plotted. (**D**) Variant calls present in all three RNA sequencing sets but not DNA were highly biased towards known patterns of RNA editing, whereas variants found in both DNA and RNA sets displayed the expected Ti: Tv behaviour. (**E**) F1 score decreases slowly as coverage is downsampled from approximately 250 M reads to 100 M, 30 M, and 5 M reads. F1 score is averaged separately for WGS or more expressed genes (TPM ≥ 2) and less expressed genes (TPM < 2), noncoding exons (NCE), or intergenic/intronic (I/I)

We examined the accuracy of RNA-seq variants, taking the DNA sequencing variants as the truth set. Although DNA-based variant calls are regarded as the gold-standard, the average depth of coverage over the 74 samples (13x) is lower than typically recommended for accurate calls (20-30x). Consequently, some false positives/negatives may be due to an imperfect truth set, particularly in heterozygous genotypes. We observed SNP precision and recall had a substantial but expected dependency on gene expression levels, with highly expressed genes (transcripts per million [TPM] ≥ 10; Supplementary Fig. 1) achieving 97.7% precision and 91.8% recall averaged across the three tissues, while genes with less than 0.1 TPM averaged 41.3% precision and 5.1% recall (Fig. 2A). Recall in genes with less than 0.1 TPM was lower than that in non-exonic regions, likely due to RNA read alignments overlapping unannotated intronic or intergenic features. Indel calling accuracy demonstrated a similar dependency on expression levels (Fig. 2B) but with overall reduced precision and recall.

We also investigated the effect of allele-specific expression (ASE) on RNA-seq variant calling. Affected RNA-seq variants show a deviation from the expected 1:1 ratio of reference and alternate alleles, which results in missed variant calls (if the alternative allele is less expressed) or incorrect homozygous alternate genotyping (if the reference allele is more expressed). We observe both these effects after excluding monoallelic expression, causing heterozygous DNA-seq variants to be missed or genotyped as homozygous alternate (Fig. 2C). Between 56 and 73% of ASE-variants were genotyped correctly, whereas extreme ASE cases (> 85% allelic imbalance) were primarily responsible for erroneous calls.

There were 960k, 1,960k, and 1,520k variants called for testis, epididymis, and vas deferens RNA-seq, respectively, which were not called by DNA-seq. We also identified 150,011 (577,839) RNA-seq variants called uniformly across all three (two) tissues but not in the DNA-seq. Given these variants occur in different, independently sampled tissues, they potentially correspond to RNA editing or other RNA modification events that are not detectable from DNA-seq and thus appear as RNA-DNA differences (RDDs) [40], rather than erroneous variant calls. Furthermore, approximately 98% of RDDs did not overlap variants from a larger panel of 501 animals of similar breeds [32], while only 8% of DNA-seq variants did not overlap, demonstrating the RDDs are not simply missed genomic variants. Indeed, genotyping errors attributable to ASE only explained approximately 6% of RDDs at heterozygous DNA-seq variants, and so are limited contributors to the overall observed error rate. The RDDs follow a highly biased distribution (Fig. 2D), suggesting a high prevalence of A-to-I editing (A→G & T→C) and to a lesser degree C-to-U editing (G→A & C→T), two commonly reported forms of post-transcriptional RNA modifications [41]. However, some of these RDDs have nearly a 100% conversion rate, suggesting this may be caused by biological mechanisms other than RNA editing or technical artefacts.

Imputing the RNA-seq variants with an external reference panel containing 501 samples [39] resulted in an overall average improvement of 10% to F1, where the 24% gain of recall was offset by a 17% drop in precision. Regions with little RNA-seq coverage, and thus sparse RNA-seq variants, had low imputation accuracy as there were insufficient markers to impute confidently (Supplementary Fig. 4). However, even in regions with dense RNA-seq variants, we still observed missed or poorly imputed variants.

The 74 total RNA-seq samples have unusually high coverage. We downsampled the RNA-seq samples to approximately 100 M, 30 M, and 5 M reads, roughly corresponding to typical sequencing depths suggested for splicing phenotypes, expression phenotypes, and low-pass analyses, and reperformed variant calling. The fraction of autosomal sequence covered by at least two RNA reads decreased sublinearly (Supplementary Fig. 2), suggesting coverage is entirely lost in lowly expressed genes but highly expressed genes are still sufficiently covered even at 5 M reads. Similarly, roughly 65%, 54%, and 23% of the autosomal sequence was within 1 Kb of an RNA variant at 100 M, 30 M, and 5 M reads (Supplementary Fig. 2). The precision of called variants decreased more quickly in non-exonic or lowly expressed regions, but the precision of variants called within moderately to highly expressed exons was minimally affected down to 30 M reads and only noticeably dropped at 5 M reads. Recall decreased slightly more rapidly than precision as coverage was reduced, but 30 M RNA reads were still enough to capture over 70% of DNA-seq variants in moderately to highly expressed exons. We also downsampled the DNA-seq samples to 100 M and 30 M reads, corresponding to genome-wide coverages of 5.3- and 1.6-fold. SNP precision and recall were slightly higher for DNA-seq at 100 M reads compared to the RNA-seq (Fig. 2e). However, at 30 M reads, the RNA-seq outperformed DNA-seq for both SNP precision and recall, although 1.6-fold DNA-seq is far below a typical variant calling depth and requires processing with low pass imputation approaches to achieve sufficiently accurate genotypes [32].

### eQTL mapping with DNA and RNA variants

**Fig. 3:**
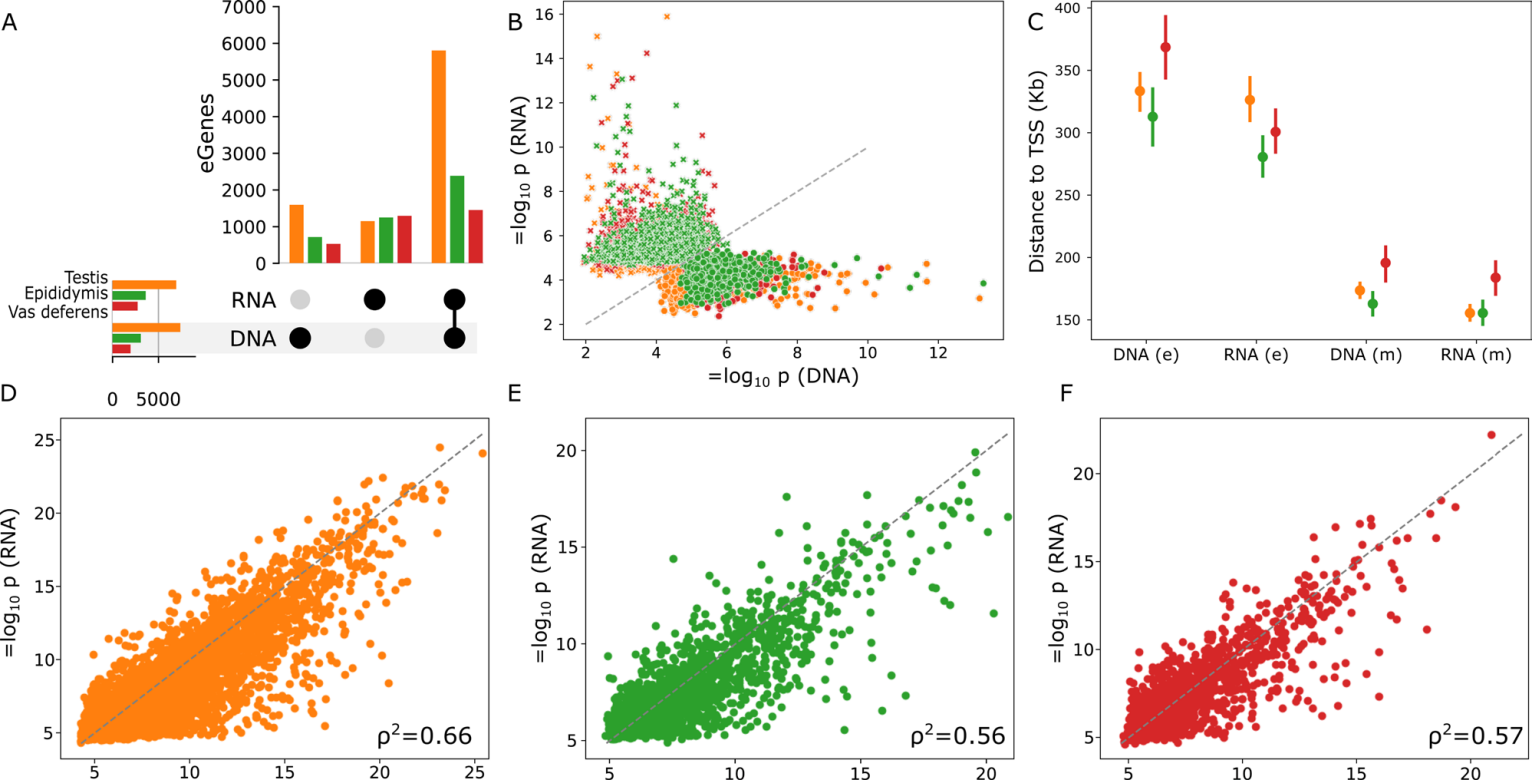
(**A**) eGenes found in both DNA and RNA variant sets or eGenes only found with RNA or DNA variants across three tissues. (**B**) The majority of eGenes found in only DNA or RNA mapping were typically close to the significance thresholds, with very few highly significant eGenes found in only one set. (**C**) eGenes found mutually (m) in both DNA and RNA sets tended to have the most significant variants closer to the TSS compared to eGenes found exclusively (e) in only one set. (**D**-**F**) P-values for the most significant variant was strongly correlated across all three tissues between the DNA and RNA variant sets for eGenes found in both

We next investigated if the quantity and quality of variants called directly from RNA-seq is sufficient to identify expression QTL (eQTL). We conducted eQTL mapping using only the RNA-seq to both genotype genomic variants and estimate gene expression. We then compared against a “truth set” which used the conventional approach of calling genomic variants from DNA-seq and estimating gene expression with RNA-seq. We ran both permutation and conditional passes to identify independent eQTL, adjusting for hidden and known covariates. We assessed significance for 20,620, 21,271, and 20,097 genes expressed in testis, epididymis, and vas deferens, respectively. The RNA-only approach was able to identify 78.9%, 77.6%, and 73.6% of genes with at least one independent-acting eQTL (eGene), respectively, compared to the DNA + RNA truth approach (Fig. 3A).

Many of the eGenes identified exclusively in either DNA- or RNA-seq variant mapping were of lower significance and close to the discovery threshold, with the other variant set (RNA- or DNA-seq respectively) typically within an order of magnitude of the significance threshold (Fig. 3B). Only 10 and 15 unique eGenes with p-values below 1 × 10^− 10^ were found in DNA- and RNA-only association mappings, respectively. Mutual eGenes found in both DNA and RNA sets were substantially closer to the transcription start site on average (Fig. 3C), as well as more significant on average compared to DNA- or RNA-only eGenes. RNA-only eGenes had substantially larger and more variable effect sizes compared to DNA-only or RNA-DNA overlapping eGenes (Supplementary Fig. 5).

For RNA-DNA overlapping eGenes, we found moderate-to-strong correlation (Spearman ρ^2^ of 0.56–0.66) of the most significant p-value for each eGene when using DNA- or RNA-seq variants (Fig. 3D-F). However, only approximately 9% of the RNA-DNA overlapping eGenes shared the same lead candidate variant, suggesting that while the significances were comparable, we rarely could recover the DNA-seq top eQTL using RNA-seq variants. The DNA-seq variants also had slightly more independent signal compared to using RNA-seq variants, although the effect was minor (1.13 versus 1.09 for testis, 1.07 versus 1.06 for vas deferens, and 1.03 versus 1.03 for epididymis).

We also conducted association mapping with the RNA-seq downsampled to 100 M, 30 M, and 5 M reads, using the reduced coverage for both the RNA-seq variants and molecular phenotypes. Due to the decrease in reads used for determining gene expression, fewer genes were expressed above filtering thresholds (Supplementary Table 2), and so fewer eQTL were identified even when using the full coverage DNA-seq variants. At 100 M RNA reads, there was minimal loss (1%) of QTL detection compared to using DNA-seq variants (Supplementary Fig. 6), and a minor loss (5%) of detection at 30 M RNA reads. Due to the substantial drop in RNA-seq variants called with 5 M reads, there was a larger loss (20%) of QTL detection at this coverage relative to using the DNA-seq variants.

### RNA DNA differences in eQTLs

**Fig. 4:**
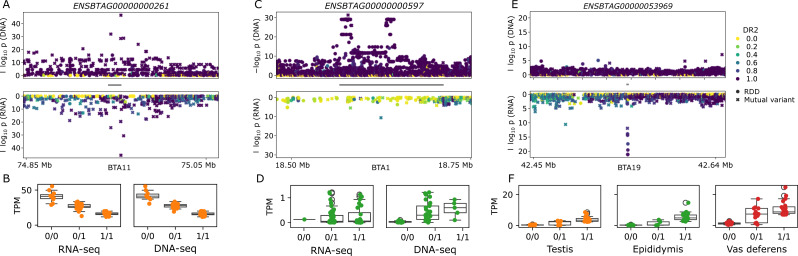
(**A**, **C**, **E**) Zoom plots for an eGene identified with both variant sets, DNA-seq variants only, and RNA-seq variants only respectively. The grey bar between the DNA and RNA associations represents the gene, while the marker colour represents imputation accuracy (DR2). The marker style indicates if the variant is present in both DNA-seq and RNA-seq variants or if it is an RDD. (**B**, **D**, **F**) TPM plots for their respective three genes. The same lead variant is used for as the genotype in **B** and **D** for testis and epididymis respectively, while the lead variant for **C** is an RDD and can only be examined for RNA-seq but is present in all three tissues

We further examined several compelling eGenes identified using only DNA- or RNA-seq variants, which typically had different distributions of RNA-seq variants and imputation accuracies compared eGenes found with both sets of variants (e.g., *ENSBTAG00000000261;* Fig. 4A, B). *ENSBTAG00000000597* was a strongly associated eGene in epididymis when using DNA-seq variants (*p* = 5.3 × 10^− 14^), but not significant with RNA-seq variants (*p* = 1.4 × 10^− 4^). The same top SNP variant was called in both DNA and RNA variant sets (Fig. 4C, D), but was poorly genotyped in epididymis RNA (allele frequency of 0.26 in DNA-seq and 0.07 in RNA-seq) resulting from a low *ENSBTAG00000000597* transcript abundance (average TPM 0.23). The poor genotyping in epididymis RNA-seq was also evident from the significant deviation from Hardy-Weinberg proportions (*p* = 9.2 × 10^− 7^) while the DNA-seq variants followed Hardy-Weinberg proportions (*p* = 0.86). Consequently, no significant association between RNA-called variants and *ENSBTAG00000000597* expression was found. Similarly, an eQTL for *ENSBTAG00000033056* was missed in testis, with only 5 low quality variants within a 5 Kb window of the lead DNA SNP. In general, almost all DNA-only eQTL were due to the lack of well genotyped RNA variants near the lead DNA SNP. We did not observe any DNA-only QTL where the missing RNA variants could be explained by ASE.

Unexpectedly, some eGenes are only identified when mapping RNA-seq variants and not with DNA-seq variants. For example, *ENSBTAG00000020116* was significant in epididymis tissue, but primarily because the significance threshold was moderately lower for RNA-seq variants. Fewer RNA-seq variants within the cis-window led to a significance threshold of 3.5 × 10^− 6^ (versus a DNA threshold of 7.9 × 10^− 7^), and the top RNA variant had *p* = 2.2 × 10^− 6^ (versus DNA top variant *p* = 5.0 × 10^− 6^). These marginal examples could be removed by setting a uniform stricter significance threshold, especially in the case of sparse variants.

Out of the 15 highly significant (*p* < 1 × 10^− 10^) RNA-seq only eGenes, only nine are annotated as protein coding, while the other six are e.g., pseudogenes or lncRNA (Supplementary Table 3). Genome-wide, protein coding genes make up 80% of the annotation, compared to only 60% of these RNA-only eGenes. Most of these highly significant RNA-seq only eGenes appear in all three examined tissues, suggesting this is not a tissue-specific observation but potentially something affecting RNA analyses more generally. Almost all these genes have multiple paralogues (Supplementary Table 3), which can lead to low-quality or ambiguous RNA alignments and thus degraded variant calling. However, we find, for example in *ENSBTAG00000053969* (Fig. 4E, F) and *ENSBTAG00000027962* (Supplementary Fig. 7), that RNA-seq coverage can be largely missing or highly expressed in a portion of the annotated exon region (Supplementary Fig. 8). The top associated variants appear within these differentially covered regions, and some homozygous reference samples have sufficient coverage to be distinguished from a missing genotype. The lack of variants in the DNA-seq and the distinct RNA coverage dropout suggest these eQTL cannot simply be explained by paralogue mismapping for the RNA reads, although it is not clear if there is an alternative artefactual explanation or a mechanism beyond the genome (e.g., RNA editing/modification, epigenome, etc.)

## Discussion

RNA sequencing is critical to examine mechanisms underpinning variation in gene expression or splicing, but its utility for variant calling had not been characterised extensively. We find deep total RNA sequencing with ~ 250 M reads covers one third of the genome, leaving many (primarily intergenic) regions inaccessible. From 74 cattle transcriptomes, we call 7-10 M variants per tissue, approximately only 40% of that from matched DNA sequencing, but still two orders of magnitude more than previously reported for cattle RNA variant calling from primarily mRNA [14]. Particularly in coding regions that are highly expressed (TPM ≥ 10), we recover over 92% of DNA-seq variants with precision of approximately 98%. Precision and recall are reduced at more typical RNA coverage levels, with 76% precision and 26% recall genome-wide at 30 M reads. Testis, epididymis, and vas deferens express substantially more genes at detectable levels compared to other tissues [4, 42], meaning that our recall values likely represent an upper bound and might be lower for most other tissues. RNA-specific effects, like allele-specific expression or RNA editing, are detrimental to variant calling accuracy but only affect a limited number of sites.

Despite total RNA-seq variant calling only capturing approximately 40% of variant sites compared to DNA-seq variant calling, it identifies roughly 75% of eGenes, and so is nearly 2-fold enriched for eQTL. This trend holds when downsampling to 30 M reads before sharply dropping at 5 M reads. Interestingly, when downsampling to 30 M reads, we find only 10–15% fewer expressed genes but roughly 50% fewer significant eGenes (Supplementary Table 2), suggesting that deep sequencing is required for comprehensively mapping eQTL [4]. The majority of eGenes identified by DNA-seq but missed by RNA-seq variants are due to eQTL being extremely distant to the TSS (> 300 Kb) or, to a lesser degree, located within lowly expressed regions leading to poor RNA-seq variant genotyping accuracy. On the other hand, highly significant eGenes unique to RNA-seq variants are mostly associated with RDDs (12 out of 15) with few variants in linkage disequilibrium which would likely fail manual curation. However, the leading RNA-only eQTL variants have high variant qualities and imputation scores (Supplementary Table 4), comparable to those in agreement with DNA-seq variant calls, and so cannot be easily filtered a priori. Furthermore, the low agreement we observed for the top associated variants between DNA-seq and RNA-seq variants would weaken downstream analyses like colocalization of putative causal variants [4] if depending only on RNA-seq variants.

Livestock GTEx consortia rely on RNA-seq for variant calling (e.g., cattle [15], chicken [17], and pig [18]) to enable molecular QTL mapping as most RNA-seq samples don’t have matched DNA-based genotypes or sequences. This is different to the equivalent human GTEx [19] which uses transcriptomes that have matched DNA whole-genome sequencing. We have comprehensively shown that RNA-seq variant calling accuracy is highly dependent on gene expression levels (~ 98% precision for TPM ≥ 10 versus ~ 75% precision for 2 > TPM ≥ 0.1) and hundreds of thousands of RDDs exist in each tissue examined. While these livestock GTExs impute RNA-seq variants into large reference panels, likely avoiding the false positive RNA-seq variant eGenes, our results demonstrate that caution is needed when using RNA-seq variants as a replacement for DNA-seq or genotyping array variants. This is especially true for regions with sparse RNA-seq variants, which remain largely inaccessible even when imputing with large reference panels. In addition, RDDs can potentially disrupt the haplotype consistency necessary for accurate imputation with large reference panels, leading to worse precision compared to just the RNA-seq variants themselves.

Considerable uncertainty remains over the origins of RDDs, and whether they are technical artefacts or biological modifications [43–45]. Over 150k variants are called in all three RNA-seq tissues but not in DNA-seq, many of which had high allele frequencies and allele depth, demonstrating that RDDs are pervasive regardless of their true origin, and cannot be simply addressed by conservative filtering. Analogous to improvements in alignment uniqueness for long read over short read DNA [46], aligning to genes with highly similar paralogues likely will likely benefit from long read RNA approaches and disentangle potential causes like paralogue or pseudogene alignments [47] for some RDDs.

## Conclusions

With recent improvements to RNA variant calling algorithms, it is possible to call millions of variants of total RNA sequencing. However, we find substantially different genotyping accuracy between highly and lowly expressed genes, as well as hundreds of thousands of high-quality RNA variants not supported by matched DNA sequencing. As such, using RNA sequencing to predict genomic genotypes may be justifiable for downstream applications, but may introduce more false positives than using DNA sequencing or genotyping arrays.

### Electronic supplementary material

Below is the link to the electronic supplementary material.

Supplementary Material 1

## Data Availability

There are no new data associated with this article, and all publicly accessible data are described in Supplementary Table 1. All scripts and pipelines used in these analyses are available at https://github.com/AnimalGenomicsETH/RNA_variant_calling.
